# First comprehensive *TSC1/TSC2* mutational analysis in Mexican patients with Tuberous Sclerosis Complex reveals numerous novel pathogenic variants

**DOI:** 10.1038/s41598-020-62759-5

**Published:** 2020-04-20

**Authors:** Miriam E. Reyna-Fabián, Nancy L. Hernández-Martínez, Miguel A. Alcántara-Ortigoza, Jorge T. Ayala-Sumuano, Sergio Enríquez-Flores, José A. Velázquez-Aragón, Alfredo Varela-Echavarría, Carlos G. Todd-Quiñones, Ariadna González-del Angel

**Affiliations:** 1 Laboratorio de Biología Molecular, Instituto Nacional de Pediatría, Secretaría de Salud, Ciudad de México, México; 2 IDIX SA de CV., Querétaro, México; 3 Grupo de Investigación en Biomoléculas y Salud Infantil, Laboratorio de Errores Innatos del Metabolismo y Tamiz, Instituto Nacional de Pediatría, Ciudad de México, México; 4 Departamento de Neurobiología del Desarrollo y Neurofisiología, Instituto de Neurobiología, Universidad Nacional Autónoma de México, Querétaro, México; 5 Posgrado en Biología Experimental, Universidad Autónoma Metropolitana-Iztapalapa, Ciudad de México, México; 6 Laboratorio de Biología Molecular, Departamento de Genética Humana, Hospital de Alta Especialidad de Veracruz, Veracruz, México

**Keywords:** Genetics, Clinical genetics, Genetic testing

## Abstract

The aim of this study was to improve knowledge of the mutational spectrum causing tuberous sclerosis complex (TSC) in a sample of Mexican patients, given the limited information available regarding this disease in Mexico and Latin America. Four different molecular techniques were implemented to identify from single nucleotide variants to large rearrangements in the *TSC1* and *TSC2* genes of 66 unrelated Mexican-descent patients that clinically fulfilled the criteria for a definitive TSC diagnosis. The mutation detection rate was 94%, *TSC2* pathogenic variants (PV) prevailed over *TSC1* PV (77% vs. 23%) and a recurrent mutation site (hotspot) was observed in *TSC1* exon 15. Interestingly, 40% of the identified mutations had not been previously reported. The wide range of novels PV made it difficult to establish any genotype-phenotype correlation, but most of the PV conditioned neurological involvement (intellectual disability and epilepsy). Our 3D protein modeling of two variants classified as likely pathogenic demonstrated that they could alter the structure and function of the hamartin (*TSC1*) or tuberin (*TSC2*) proteins. Molecular analyses of parents and first-degree affected family members of the index cases enabled us to distinguish familial (18%) from sporadic (82%) cases and to identify one case of apparent gonadal mosaicism.

## Introduction

Tuberous sclerosis complex (TSC; MIM #191100, MIM #613254) is an autosomal dominant syndrome characterized by the presence of multiple hamartomas in different organs and systems. The incidence is about 0.1–1/10,000 births and the prevalence varies from 1/6,000 to 1/10,000 among different populations^1–3^. The manifestations of TSC are highly variable among individuals and even within the same family^4^, but the most common clinical features are localized in skin and central nervous system^5–7^.

TSC is caused by pathogenic variants (PV) in the tumor suppressor-genes, *TSC1* (tuberous sclerosis complex 1, MIM *605284, 9q34.13) and *TSC2* (tuberous sclerosis complex 2, MIM *191092, 16p13.3). These PV can be detected by various molecular techniques, such as single-strand conformational polymorphism (SSCP), direct Sanger sequencing (SS), multiplex ligation-dependent probe amplification (MLPA) and next-generation sequencing (NGS). There are currently more than 2,000 pathogenic *TSC1*/*TSC2* variants described in the Leiden Open Variation Database (www.lovd.nl/TSC1 and www.lovd.nl/TSC2)^8^; of them, 21–26% are located in *TSC1* and 69–79% in *TSC2*^9,10^. In approximately 5–25% of the analyzed TSC cases, a PV could not be identified in either gene^9,11,12^. The emergence of new techniques, such as NGS, has significantly increased the mutation detection rate in cases where conventional tests were not successful (i.e., by identifying low rate somatic mosaic variants)^11–13^, but it remains difficult to establish any phenotype-genotype correlation in TSC. It has been proposed that the more severe phenotypes (in terms of the quantity or severity of the clinical features) are mainly *TSC2-*related. Certain other clinical manifestations, including subependymal giant cell astrocytoma (SEGA), renal angiomyolipomas and cardiac rhabdomyomas, are more common in patients with *TSC2* variants^14–16^.

Most of the studies on TSC have been implemented in populations of Europe, USA, Canada and Brazil; hence, it is relevant to include more cases from other Latin American countries in order to investigate and expand the responsible TSC genotype, better delineate the clinical features and contribute to possibly identifying genotype-phenotype correlations. To our knowledge, there are only two previous published molecular studies of TSC patients in Mexico: one involving three patients with early-stage polycystic kidney disease that were molecularly confirmed to represent *TSC2/PKD1* contiguous gene syndrome cases^17^ and one involving three *TSC2*-cases with prenatally documented cardiac rhabdomyomas^18^. Therefore, in order to improve our knowledge of this disease and to spread the use of innovative and highly sensitive molecular techniques such as MLPA and NGS for the diagnosis of TSC in countries where the disease has been under-studied, we used a combined molecular strategy to analyze the mutational spectrum of *TSC1* and *TSC2* and the principal clinical features of 66 Mexican-descent unrelated cases of TSC.

## Results

### Patients

This study included 66 unrelated patients recruited between 2008 and 2017 in the genetics service at the National Institute of Pediatrics in Mexico City, Mexico. All patients were clinically classified as definitive TSC cases according to the most recent diagnostic criteria^19^. Thirty-six cases were male (55%) and 30 were female (45%), and the mean age at diagnosis was 6 years 6 months (range: 1 month – 24 years of age) with a median age of 6 years. Most patients were diagnosed during childhood (*N* = 36, 55%); the rest were diagnosed in infancy and adolescence (*N* = 15, 23%; *N* = 14, 21% respectively), and only one in adulthood (*N* = 1, 1.5%).

Clinical evaluation by a medical geneticist and imaging studies (cranial computed tomography and renal ultrasonography) on available parents and potentially affected family members from the 66 index cases allowed us to classify 54 cases as sporadic (without any family history of TSC) and 12 cases as familial (with one or more affected members). One of the familial cases was considered to represent possible gonadal mosaicism (two siblings were affected while both parents were healthy).

### Mutational analyses of *TSC1* and *TSC2*

#### SSCP and SS

**Figure 1:**
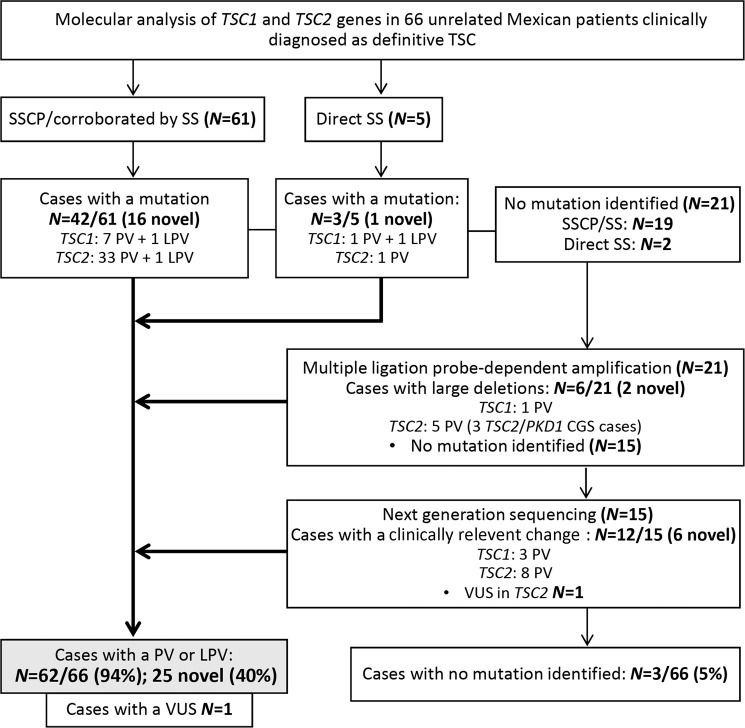
Molecular algorithm used in 66 definitive TSC patients. Abbreviations: CGS: *TSC2-PKD1* contiguous gene syndrome; LPV: likely pathogenic variant; PV: pathogenic variant; SS: Sanger sequencing; SSCP: single-strand conformation polymorphism; VUS: variant of uncertain significance.

Genomic DNA samples derived from all 66 cases were initially subjected to mutational analyses of *TSC1* and *TSC2* by a SSCP assay followed by SS confirmation in 61 cases and direct SS in the remaining five cases (Fig. 1). Both assays included all coding and non-coding (20 bp at the exon-intron boundaries) regions of the *TSC1* (NM_000368.4) and *TSC2* (NM_000548.3) genes. These analyses identified a clear disease-causing PV in 40/61 cases studied by SSCP/SS and in two of five cases studied by direct SS (Table 1). Three other variants were classified as likely pathogenic variants (LPV) according to guidelines of the American College of Medical Genetics and Genomics and the Association for Molecular Pathology (ACMG/AMP)^20^. The two LPV in *TSC1* were c.737+3A>G and p.(Leu112_Leu113delinsLysGluVal) from cases ET75 and ET201, respectively, and the single in-frame LPV in *TSC2*: p.(His1746_Arg1751dup) from case ET171. From these three cases, solely in case ET75, analysis of the proband’s paternity and maternity (criterion PS2)^20^ using 15 short tandem repeat markers (13 of them belong to the CODIS system) could be performed and confirmed parentage, but this ACMG/AMP criterion was not enough to re-classify the LPV as pathogenic (Table 2). Therefore, our analysis identified a PV or LPV in 42/61 cases studied by SSCP/SS and in three of five cases studied by direct SS (Fig. 1).

**Table 1:** General information for the 63 TSC patients in whom we identified a pathogenic variant (PV), likely pathogenic variant (LPV) or variant of unknown significance (VUS).

*TSC1 gene*									
Location	Nucleotide change (NM_000368.4)	Codon change (NP_000359.1)	Clinical significance^a^	Molecular technique	Inheritance^b^	Previous reports LOVD/dbSNP /ExAC/gnomAD/ClinVar/HGMD/Literature	Case	Sex	Age♦
Exon 3	c.89_102del	p.(Lys30Ilefs*2)	Pathogenic	SSCP	Familial (mother and brother heterozygous)	**NPR (LOVD: TSC1_001334)**	ET173	M	12 y
Exon 5	c.333_337delinsAAAAGAGG	p.(Leu112_Leu113delinsLysGluVal)	**Likely Pathogenic**	SSCP	suspected de novo	**NPR (LOVD: TSC1_001335)**	ET201	M	7 y
Exon 8	c.682C>T	p.(Arg228*)	Pathogenic	SSCP	de novo	TSC1_000037 (+/+)/rs118203427/NR/NR/49083/CM981931	ET25	M	5 y
Intron 8	c.737+3A>G	—	**Likely Pathogenic**	SS	de novo	TSC1_000041 (-/+?)/rs118203439/NR/NR/49093/NR	ET75	M	10 y
Exon 15	c.1458_1461del	p.(Ser487Argfs*44)	Pathogenic	SS	suspected de novo	**NPR (LOVD: TSC1_001336)**	ET157	M	6 y
Exon 15	c.1888_1891del	p.(Lys630Glnfs*22)	Pathogenic	SSCP	Familial (mother and brother heterozygous)	TSC1_000116 (+/+)/rs118203595/NR/NR/5097/CD972488	ET93	F	2 y
Exon 15	c.1888_1891del	p.(Lys630Glnfs*22)	Pathogenic	SSCP	de novo	TSC1_000116(+/+)/rs118203595/NR/NR/5097/CD972488	ET249	M	10 y
Exon 15	c.1959dup	p.(Gln654Thrfs*34)	Pathogenic	NGS	Familial (daughter heterozygous)	TSC1_000121 (+/+)/rs118203603/NR/NR/48857/CI067260	ET264	M	16 y
Exon 17	c.2101C>T	p.(Gln701*)	Pathogenic	NGS	de novo	**NPR (LOVD: TSC1_000876)**	ET107	M	2 y
Exon 18	c.2227C>T	p.(Gln743*)	Pathogenic	NGS	Familial (father and sister heterozygous)	TSC1_000145(+/+)/rs118203661/NR/NR/48921/CM971522	ET190	M	4 y
Exon 18	c.2341C>T	p.(Gln781*)	Pathogenic	SSCP	Familial (father and sister heterozygous)	TSC1_000155 (+/+)/rs118203680/NR/NR/48941/CM052373	ET130	F	4Y
Exon 18	c.2356C>T	p.(Arg786*)	Pathogenic	SSCP	No parental DNA for testing	TSC1_000156(+/+)/rs118203682/NR/NR/48943/CM971523	ET213	M	24 y
Exon 20	c.2596_2600dup	p.(Gln867Hisfs*13)	Pathogenic	SSCP	de novo	**NPR (LOVD: TSC1_001338)**	ET117	M	9 y
Exon 15–23	c.(1439+1_1997-1)_(2976 + 1_*4888)del	—	Pathogenic	MLPA	de novo	**NPR (LOVD: TSC1_001339)**	ET254	F	7 y
***TSC2 gene***									
**Location**	**Nucleotide change (NM_000548.4)**	**Codon change (NP_000539.2)**	**Clinical significance**^**a**^	**Molecular technique**	**Inheritance**^**b**^	**Previous reports LOVD/dbSNP/ExAC/gnomAD/ClinVar/HGMD/Literature**	**Case**	**Sex**	**Age**
Intron 5	c.481+5G>T	—	Pathogenic	SSCP	de novo	TSC2_000966 (+/+?)/rs137854135/NR/NR/49825/NR/Tybuczy *et al*., 2015^12^	ET96	F	6 y
Exon 8	c.668dup	p.(Asp223Glufs*12)	Pathogenic	SSCP	suspected de novo	**NPR (LOVD: TSC2_004257)**	ET166	M	6 y
Exon 10	c.912G>A	p.(Trp304*)	Pathogenic	SSCP	Familial (father heterozygous)	TSC2_001218 (+/+)/rs397514884/NR/NR/64852/CM010495	ET236	M	8 y
Intron 12	c.1258-1G>C	—	Pathogenic	SSCP	de novo	**NPR (LOVD: TSC2_004258)**	ET41	M	10 y
Intron 12	c.1258-2A>G	—	Pathogenic	SSCP	No parental DNA for testing	**NPR (LOVD: TSC2_002492)**	ET200	M	1 m
Exon 17	c.1831C>T	p.(Arg611Trp)	Pathogenic	SSCP	de novo	TSC2_000053(+/+)/rs45469298/NR/NR/49643/CM961387	ET161	F	3 y
Exon 17	c.1832G>A	p.(Arg611Gln)	Pathogenic	SSCP	de novo	TSC2_000105 (+/+)/rs28934872/NR/NR/12397/CM981945	ET72	M	1 y
Exon 18	c.1841C>A	p.(Ala614Asp)	Pathogenic	SSCP	suspected de novo	TSC2_000188 (+/+?)(+?/+?)/rs45454398/NR/NR/49721/CM991204	ET120	M	9 y
Exon 18	c.1841C>A	p.(Ala614Asp)	Pathogenic	SSCP	de novo	TSC2_000188(+/+?)(+?/+?)/rs45454398/NR/NR/49721/CM991204	ET148	F	8 m
Exon 18	c.1881_1882dup	p.(Arg628Profs*71)	Pathogenic	NGS	de novo	**NPR (LOVD: TSC2_004259)**	ET32	F	1 y
Intron 19	c.2098-1G>A	—	Pathogenic	SSCP	suspected de novo	TSC2_000439(+/+)/rs45517212/NR/NR/49730/CS010577	ET232	F	9 y
Exon 20	c.2172dup	p.(Thr725Tyrfs*37)	Pathogenic	SSCP	de novo	**NPR (LOVD: TSC2_004260)**	ET238	M	3 y
Exon 21	c.2309_2315del	p.(Leu770Hisfs*2)	Pathogenic	SSCP	de novo	**NPR (LOVD: TSC2_004261)**	ET53	F	8 m
Exon 22	c.2448dup	p.(Asp817*)	Pathogenic	NGS	suspected de novo	**NPR (LOVD: TSC2_004262)**	ET122	F	3 y
Intron 23	c.2640-1G>T	—	Pathogenic	SSCP	suspected de novo	**NPR (LOVD: TSC2_004263)**	ET159	F	2 y
Exon 27	c.3094C>T	p.(Arg1032*)	Pathogenic	SS	suspected de novo	TSC2_000492 (+/+)/rs45465195/NR/NR/49240/CM001801	ET277	M	9 m
Exon 28	c.3134_3136delinsTTTT	p.(Ser1045Phefs*123)	Pathogenic	NGS	suspected de novo	**NPR (LOVD: TSC2_004264)**	ET278	F	7 m
Exon 28	c.3179G>C	p.(Trp1060Ser)	Pathogenic	NGS	de novo	**NPR (LOVD: TSC2_004265)**	ET243	M	5 y
Exon 28	c.3277G>T	p.(Glu1093*)	Pathogenic	SSCP	de novo	**NPR (LOVD: TSC2_004266)**	ET87	M	4 y
Exon 29	c.3371_3381del	p.(Ala1124Glyfs*40)	Pathogenic	SSCP	Familial (father and cousin from the father´s side heterozygous)	**NPR (LOVD: TSC2_004267)**	ET175	M	11 y
Exon 30	c.3532C>T	p.(Gln1178*)	Pathogenic	SSCP	de novo	TSC2_000269(+/+)/NR/NR/NR/49263/CM992688	ET22	F	12 y
Exon 30	c.3538A>T	p.(Lys1180*)	Pathogenic	SSCP	Familial (mother heterozygous)	**NPR (LOVD: TSC2_004268)**	ET145	F	2 m
Exon 31	c.3624G>A	p.(Trp1208*)	Pathogenic	SSCP	Familial (brother heterozygous); gonadal mosaicism	**NPR (LOVD: TSC2_002982)**	ET28	F	14 y
Exon 34	c.4174C>T	p.(Gln1392*)	Pathogenic	SSCP	de novo	TSC2_000563 (+/+)/rs45517330/NR/NR/49806/CM091103	ET124	F	17 y
Exon 34	c.4180_4181delCT	p.(Leu1394Alafs*19)	Pathogenic	SSCP	suspected de novo	TSC2_000565 (+/+)/rs137854363/NR/NR/50061/NR	ET56	M	6 m
Exon 34	c.4318C>T	p.(Gln1440*)	Pathogenic	NGS	de novo	TSC2_000860 (+/+)/rs45517337/NR/NR/49524/CM078630	ET241	M	14 y
Exon 34	c.4367_4385del	p.(Leu1456Profs*14)	Pathogenic	SSCP	Familial (father heterozygous)	**NPR (LOVD: TSC2_004270)**	ET168	M	6 y
Exon 34	c.4375C>T	p.(Arg1459*)	Pathogenic	SSCP	Familial (mother heterozygous)	TSC2_000221 (+/+)/rs45517340/NR/rs45517340/49986/CM991214	ET188	F	17 y
Exon 35	c.4496dup	p.(Val1500Argfs*24)	Pathogenic	SSCP	de novo	TSC2_002387 (+/+)/rs397515194/NR/NR/65267/NR	ET35	M	3 y
Exon 35	c.4560del	p.(Asn1522Metfs*54)	Pathogenic	SSCP	de novo	**NPR (LOVD: TSC2_004271)**	ET16	F	9 y
Exon 36	c.4581del	p.(Phe1527Leufs*49)	Pathogenic	NGS	de novo	**NPR (LOVD: TSC2_004272)**	ET146	M	1 y
Exon 36	c.4620C>A	p.(Tyr1540*)	Pathogenic	SSCP	de novo	TSC2_000595(+/+)/rs45455897/NR/NR/49263/CM091132	ET19	F	12 y
Exon 36	c.4660C>T	p.(Gln1554*)	Pathogenic	SSCP	suspected de novo	TSC2_002901 (+/+)/NR/NR/NR/NR/NR	ET195	F	8 y
Exon 37	c.4830G>A	p.(Trp1610*)	Pathogenic	SSCP	de novo	TSC2_000615 (+/+)/rs45517372/NR/NR/49841/CM091137	ET127	F	9 m
Intron 37	c.4849+2_4849+11del	—	Pathogenic	SSCP	suspected de novo	**NPR (LOVD: TSC2_004273)**	ET114	M	3 y
Exon 38	c.4918C>T	p.(His1640Tyr)	Pathogenic♣	NGS	suspected de novo	TSC2_000598 (+/+?)/rs45485092/NR/NR/49333/CM090851/Coevoets *et al*., 2009^60^	ET7	F	12 y
Exon 39	c.5024C>T	p.(Pro1675Leu)	Pathogenic	SSCP	de novo	TSC2_000033(+/+)/rs45483392/NR/NR/12393/CM971532	ET66	F	2 y
Exon 39	c.5024C>T	p.(Pro1675Leu)	Pathogenic	SSCP	de novo	TSC2_000033(+/+)/rs45483392/NR/NR/12393/CM971532	ET154	M	8 y
Intron 40	c.5160+5G>T	—	Pathogenic♣	NGS	de novo	TSC2_000651 (+/+)(+?/+)/rs45515392/NR/NR/49430/CS091153/Avgeris *et al*., 2017^32^	ET4	F	6 y
Exon 41	c.5238_5255del	p.(His1746_Arg1751del)	Pathogenic	SSCP	de novo	TSC2_000149 (+/+)/rs137854218/NR/NR/12402/CD982991	ET139	M	20 y
Exon 41	c.5238_5255del	p.(His1746_Arg1751del)	Pathogenic	SSCP	de novo	TSC2_000149 (+/+)/rs137854218/NR/NR/12402/CD982991	ET142	F	1 y
Exon 41	c.5238_5255del	p.(His1746_Arg1751del)	Pathogenic	SSCP	de novo	TSC2_000149 (+/+)/rs137854218/NR/NR/12402/CD982991	ET151	F	5 y
Exon 41	c.5238_5255dup	p.(His1746_Arg1751dup)	**Likely Pathogenic**	SSCP	suspected de novo	**TSC2_004274**/rs137854218/NR/rs137854218/NR/NR	ET171	M	7 m
Exon 1—15	Deletion exons 1-15 NG_005895.1(NM_000548.4):c.(?_−106)_(1444 + 1_1599-1)del GRCh38 Chr16 NC_000016:g.(?_2047464)_(2064272-2064427)		Pathogenic	MLPA	de novo	TSC2_001076 (+/+)(+?/+)/NR/NR/NR/NR/CG015688,CG015689	ET104	M	1 m
Exon 17—36	Deletion exons 17-36 NM_000548.4: c.(1716 + 1_1717-1)_(4662 + 1_4663-1)del NC_000016: g(2070456_2085322)del (GRCh38)		Pathogenic	MLPA	Familial (mother and brother heterozygous)	**NPR (LOVD: TSC2_004276)**	ET90	F	13 y
Exon 1—42	Complete TSC2 deletion + PKD1 (Exons 20-46) arr[hg38] 16p13.3(1,875,332-2,106,147)x1/HS3ST6, MSRB1, RPL3L, NDUFB10, RPS2, RNF151, NOXO1, GFER, SYNGR3, ZNF598, NPW, NTHL,SLC9A3R2.		Pathogenic	MLPA CMA	de novo	NR/NR/NR/NR/NR/NR/Reyna-Fabián *et al*., 2019^17^	ET178	M	3.5 y
Exon 31—42	DeletionTSC2 (Exons 31-42) + PKD1 (Ex 46-40) NG_005895.1(NM_000548.4):c.(3610 + 1_3611-1)_(5260_*102)del NG_008617.1(NM_001009944.2):c.(?_11411)_(12445_*1017)del		Pathogenic	MLPA	suspected de novo	NR/NR/NR/NR/NR/NR/Reyna-Fabián *et al*., 2019^17^	ET183	M	7 m
Exon 31—42	DeletionTSC2 (Exons 31-42) + PKD1 (Ex 46-40) NG_005895.1(NM_000548.4):c.(3610 + 1_3611-1)_(5260_*102)del NG_008617.1(NM_001009944.2):c.(?_11411)_(12445_*1017)del		Pathogenic	MLPA	de novo	NR/NR/NR/NR/NR/NR/Reyna-Fabián *et al*., 2019^17^	ET1	F	17 y
Intron 31	c.3815-21G>A	—	VUS	NGS	suspected de novo	**TSC2_004269**/rs778201014/A = 0.0002/19/NR/rs778201014/NR/NR	ET81	M	8 y

Symbols: ^a^classified according to ACMG/AMP criteria^20^; ^b^assigned by molecular study of parents (if available) ♦ age at diagnosis; ♣ variant effect assigned by functional studies. Abbreviations: CMA: chromosomal microarray analysis; F: female; m: months; M: male; MLPA: multiplex ligation-probe amplification; NGS: next-generation sequencing; NPR: not previously reported in any public Database or literature; NR: not reported; SS: Sanger sequencing; SSCP: single-strand conformation polymorphism; y: years.

**Table 2:** Molecular information and *in silico* evaluation of the three LPV and one VUS.

**(A) not previously reported variants**											
Case	Inheritance	Gene	Location	Identified Variant	Clinical significance^†^	*In silico* analyses					
						PROVEAN Score^1^	Mutation Taster^2^				
201^a^	suspected *de novo* (father not studied)	*TSC1*	Exon 5	c.333_337delinsAAAAGAGGp.(Leu112_Leu113delinsLysGluVal)	Likely pathogenic (V) [PM2, PM5, PP3, PP4]	−11.94 deleterious	0.9989 disease causing				
**(B) variants previously reported in public Databases**											
Case	Inheritance	Gene	Location	Identified Variant	Clinical significance^†^	*In silico* analyses					GnomAd database,
						Splice Site Finder	MaxEntScan	NNSPLICE	GeneSplicer	PROVEAN Score^1^	Allele frequency Total/Latino
75	*de novo*	*TSC1*	Intron 8	c.737+3 A>G	Likely pathogenic (II) [PM2, PS2, PP3, PP4]	Natural donor splicing site abolished	Drastically diminishing (−74.7%) natural donor splicing site recognition	Natural donor splicing site abolished	Natural donor splicing site abolished	—	rs118203439, no data available
171^a^	suspected *de novo* (father not studied)	*TSC2*	Exon 41	c.5238_5255dupp.(His1746_Arg1751dup)	Likely pathogenic (IV) [PM1, PM2, PM4, PP3, PP4]	—	—	—	—	−9.222 deleterious	rs1236719116, 0.000003998/0.000
81	suspected *de novo*	*TSC2*	Intron 32	c.3815-21 G > A	Variant of uncertain significance [PP3, PP4]	No change	No change	Slight decrease (−6.9%) in recognition of natural acceptor splicing site	Strengthened recognition (35%) for natural acceptor splicing site	—	rs778201014, 0.0001279/0.0009600

Symbols: ^1^ value < −2.5 is deleterious; ^2^ value close to 1 indicates a high ‘security’ of the prediction; ^a^protein modeling was performed in these variants; ^†^classified according to ACMG/AMP criteria^20^.

#### MLPA to identify deletions or duplications

**Figure 2:**
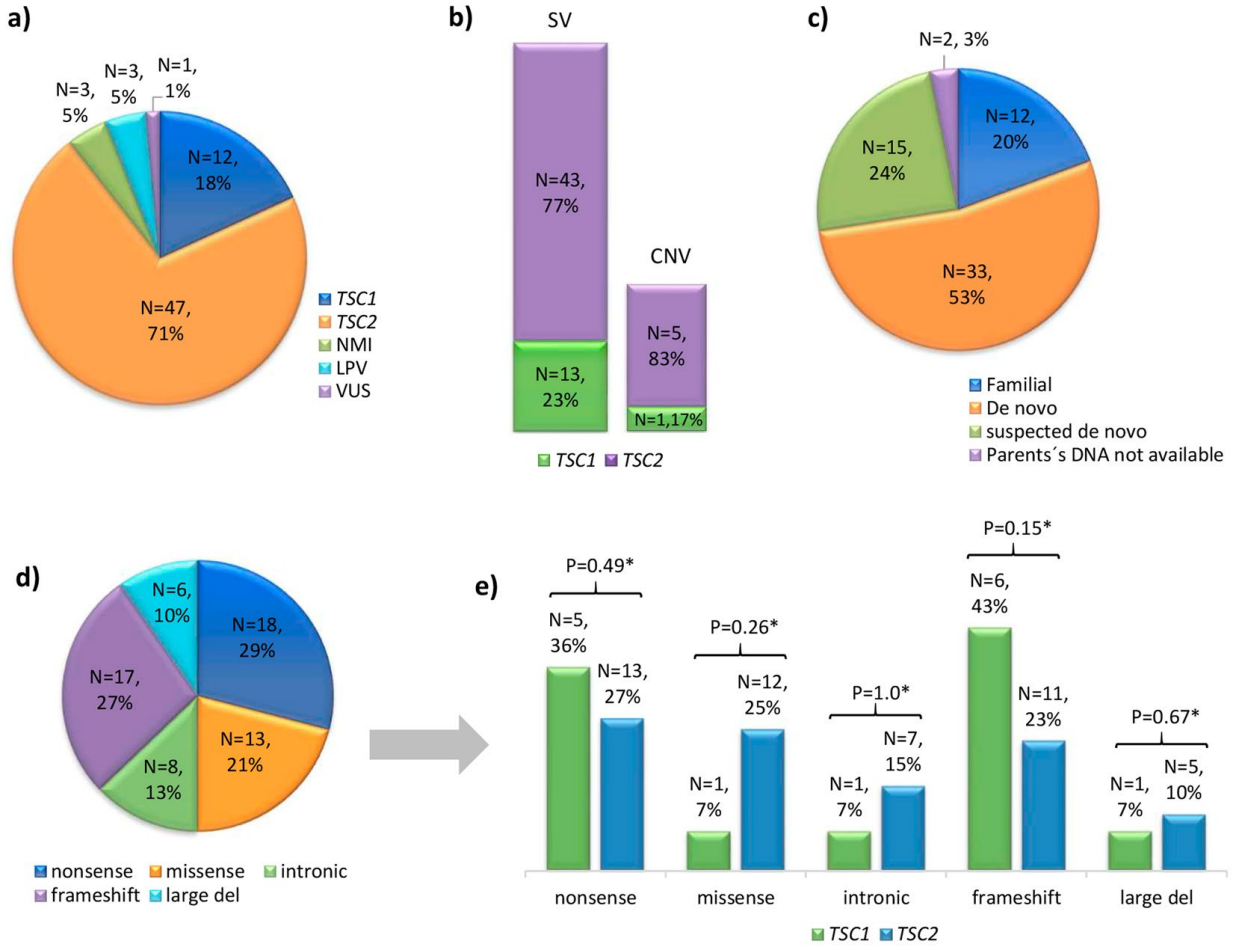
Overview of the mutational spectra in the *TSC1* and *TSC2* genes. (**a**) Numbers of cases with: PV, LPV, VUS or no mutation identified (NMI) in *TSC1* or *TSC2*. (**b**) proportion of small variants (SV) and large deletions (Copy number variant; CNV) in *TSC1* and *TSC2*. (**c**) Number of familial, *de novo* or suspected *de novo* cases assigned by molecular study of the parents (when available). (**d**) Mutation types among the 62 studied cases in which a PV or LPV was identified in either gene. (**e**) Proportions of each type of mutation in *TSC1* vs. *TSC2*; *indicates that no significant difference (p > 0.05) was found.

**Figure 3:**
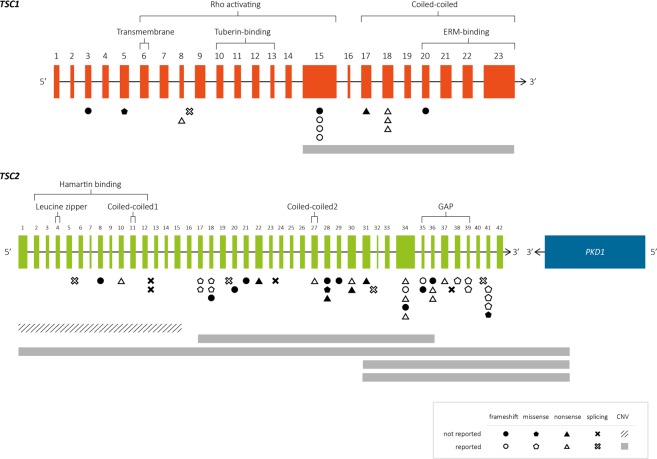
Overview of the genetic distribution of PV, LPV and VUS identified in *TSC1* (n = 14) and *TSC2*. (n = 49) Exons (orange and green boxes) and introns (gray lines) are not drawn to scale. The information above the brackets depict the domains of hamartin and tuberin according to Rosner *et al*., 2004^61^, Vries and Howe 2007^62^ and Knowles *et al*., 2009^63^. Below, the vertical gray and dotted lines indicate the range of each heterozygous deletion. Abbreviations. ERM: ezrin, radixin, moesin; GAP: GTPase-activating protein; UTR: untranslated region.

In order to identify copy number variants (CNV) at both genes in the remaining 21 cases, we performed MLPA (Fig. 1). The utilized *TSC2* probemix contained one probe (exon 40) for the *TSC2*-adjacent gene, *PKD1* (polycystic kidney disease 1, MIM *601313), whose mutation cause autosomal dominant polycystic kidney disease (ADPKD, MIM#173900). Our MLPA results identified six heterozygous gross deletions: one in *TSC1* and five in *TSC2 (*Table 1, Fig. 2b). Of the five gross deletions in *TSC2*, three involved at least exons 40–46 of *PKD1* (Fig. 3) resulting in the *TSC2-PKD1* contiguous gene syndrome (CGS, MIM #600273), which were previously published^17^. Interestingly, one of those *TSC2-PKD1* CGS cases (ET178) showed an atypical mild polycystic kidney phenotype.

#### Next-generation sequencing

Finally, an NGS study examining *TSC1* and *TSC2* coding exons and intron-exon boundaries (150 bp) was carried out in the remaining 15 cases (Fig. 1). The median depth of coverage was 639×(range 86×–1940×) with a 99.9% width of coverage. A customized bioinformatic analysis enabled us to identify a PV in 10 cases; we also found one case (ET243) with a missense variant p.(Trp1060Ser) in *TSC2* that was classified as an LPV^20^ and one case (ET81) with an intronic variant c.3815–21G>A in *TSC1* that was classified [PP3, PP4]^20^ as a variant of uncertain significance or VUS (Tables 1,2). All NGS-identified variants were confirmed by SS in the index cases and their available parents. As the missense *TSC2* p.(Trp1060Ser) LPV from case ET243 was not reported in the main genotype databases and we did not find it in 212 alleles of healthy and ethnically matched individuals assessed by a specific-allele PCR assay (data not shown), we were able to re-classify it as a pathogenic variant (IIIa) [PM2, PS2, PS4, PP3, PP4]^20^. In the remaining three cases (ET44, ET61 and ET223), no mutation was identified (NMI; lacking any LPV, VUS or pathogenic genotype) by the implemented molecular technologies (Fig. 1).

To summarize, we were able to identify a PV or LPV in 62 cases and we could not identify a PV or LPV in four cases, although one of them (case ET81) was found to harbor a VUS in *TSC2* (c.3815–21G>A) (Figs. 2a and 3). Of the identified changes, 56 (90%) corresponded to small variants (SV) such as point mutations, deletions, small insertions/deletions (InDels) and duplications, while six were large deletions (10%). Far more of the identified changes were found in *TSC2* (*N* = 48) than *TSC1* (*N* = 14, Fig. 2b). The mutational proportions for *TSC1* and *TSC2* are shown in Fig. 2d,e. Eight intronic variants were identified at both genes; five affected canonical splice sites (in *TSC2*) and three affected intronic splicing enhancers sequences (*TSC1*: c.737+3A>G; *TSC2*: c.481+5G>T and c.5160+5G>T).

Based on our review of the literature and public databases, including the Leiden Open Variation Database (LOVD, www.lovd.nl/), dbSNP (https://www.ncbi.nlm.nih.gov/projects/SNP), Exome Aggregation Consortium (http://exac.broadinstitute.org), Genome Aggregation Database (gnomad.broadinstitute.org/), ClinVar (https://www.ncbi.nlm.nih.gov/clinvar/), and Human Genome Mutation Database (http://www.hgmd.cf.ac.uk/), we determined that 25 of the 62 (40%) PV or LPV identified herein (six in *TSC1* and 19 in TSC2) had not been previously reported. All of them have been submitted to LOVD (Table 1). Of these 25 novel variants, 24 were considered pathogenic and one was an LPV^20^ in *TSC1* [p.(Leu112_Leu113delinsLysGluVal)].

Direct molecular screening in parents (when available) of the 62 cases with a PV/LPV showed that the pathogenic allele was absent from both parents for 33 patients (*de novo* cases). However, in 12 cases with one or more clinically affected family members, we confirmed the same PV in the available affected cases (familial cases, see Table 1). We suspect gonadal mosaicism in familial case ET28 as we identified a novel heterozygous PV in *TSC2*: c.3624G>A or p.(Trp1208*) in two affected siblings but failed to find this allele in peripheral blood leukocyte DNA of both clinical healthy parents. This argument was further strengthened when we confirmed the proband’s paternity and maternity by DNA profiling (data not shown). In 15 cases, we could not analyze the father’s DNA but there was no reported family history of TSC, so we designated these as suspected *de novo* cases. In the remaining two cases, the mother’s and father’s DNA samples were not available for testing (Table 1, Fig. 2c).

### Protein modeling of two missense LPV

**Figure 4:**
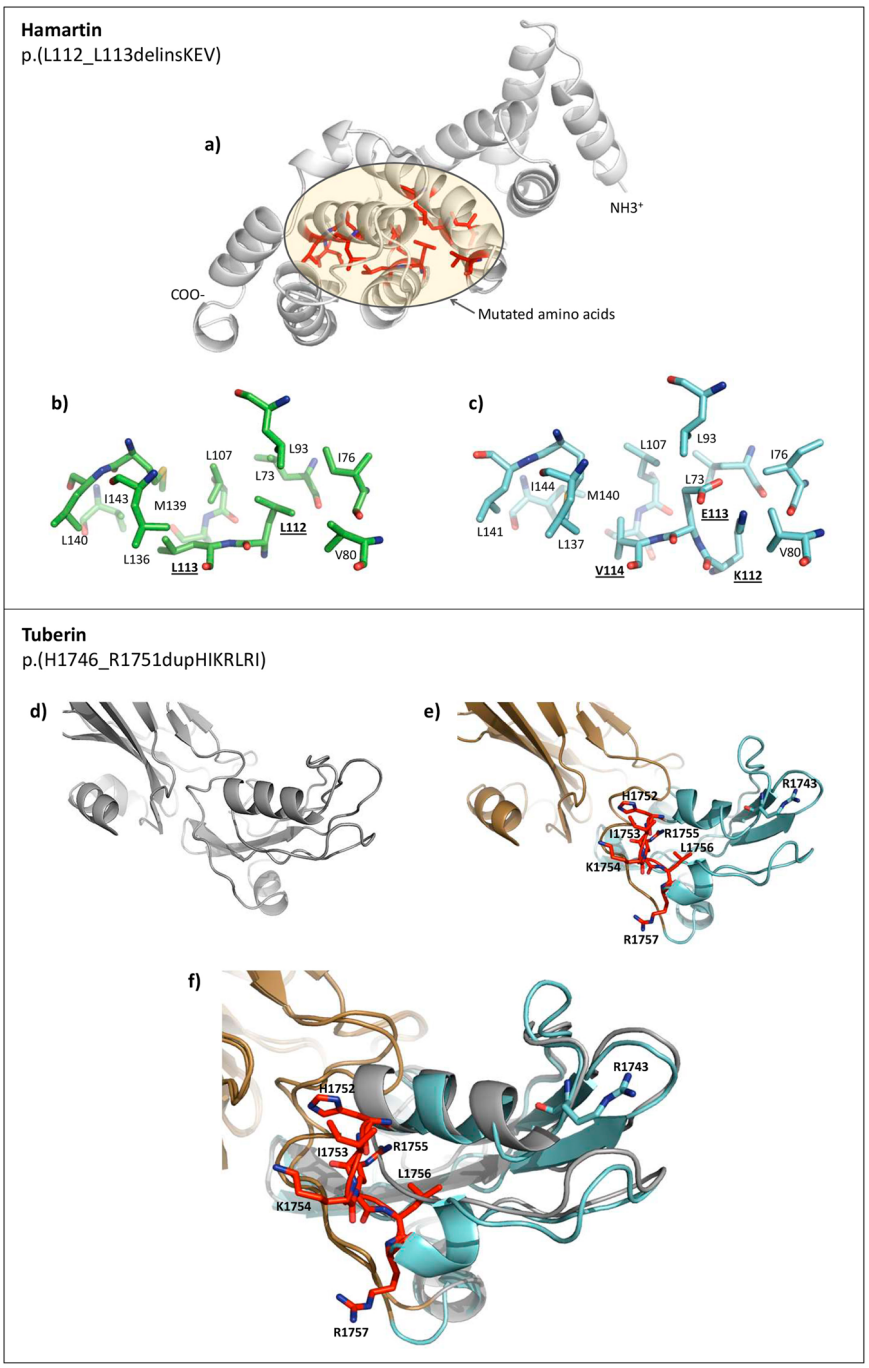
Schematic representations of the modeled N-term and C-term regions of hamartin and tuberin, respectively. (**a**) Wild-type (WT) hamartin protein. In red it shows the mutated (MUT) protein region and other amino acid residues close to the impacted zone. Yellow circle represents the hydrophobic zone and mutated amino acids. (**b,c**) Zoomed images of the mutated zones in WT and MUT hamartin, respectively. In MUT, K-112, E-113 and V-114 (bolded and underlined) are inserted. The mutated amino acids are predominantly adjacent hydrophobic amino acids. (**d,e**) The C-term regions of WT and MUT tuberin, respectively. In MUT, the side chain of the inserted amino acids (red) and the RAB domain (brown) are shown. (**f**) Zoomed image of the mutated zone. WT and MUT were superposed and compared at the secondary structure level. Conformational differences are observed, principally in the duplication zone (IKRLRH in red). Notably, the inserted amino acids are close to the RAB domain (brown). The models were generated with PyMOL^59^.

To examine possible functional and structural consequences of the two in-frame variants that were classified as LPV [*TSC1*: c.333_337delinsAAAAGAGG or p.(Leu112_L113delinsLysGluVal) and *TSC2*: c.5238_5255dup or p.(His1746_Arg1751dup)], we modeled the protein structures of the N-terminal region of wild-type (WT) and mutated (MUT) p.(Leu112_L113delinsLysGluVal) hamartin variant and the C-terminal region of WT and MUT p.(His1746_Arg1751dup) tuberin variant (Fig. 4). The modeled hamartin WT and MUT 3D structures showed that the amino acid residues surrounding the insertion/deletion region have a hydrophobic character in the WT protein, and the insertion of Lys112Glu113Val114 (two of which are ionizable) could alter the stability of this hydrophobic region. Previous work showed that the incorporation of negatively charged residues in proteins with hydrophobic clusters can provoke a significant structural alteration, and that such residues are therefore usually excluded from hydrophobic pockets^21^. Our modeling of tuberin revealed that the six duplicated amino acid residues (HisIleLysArgLeuArg at positions 1752–1757) drastically altered the secondary structure of the C-terminal end region of the MUT protein compared to the WT protein (Fig. 4f). The mutated region was found to lie in close contact with the GAP domain, suggesting that the inserted amino acids could significantly alter the GAP domain contacts. Notably, the inserted amino acids are located close to Arg1743 in the primary sequence, and a previous report showed that the Pro1743 mutation can abolish the GAP activity of tuberin^22^. Hence, this region seems to be critical for the correct function of tuberin.

### Clinical manifestations in patients with novel genetic variants

We identified novel PV in 24 TSC cases and had detailed TSC clinical information for 22 of them (see Supplementary Table S1). We were not able to identify a clear phenotype-genotype correlation since each variant was unique. However, if we exclude the single neonatal patient ET200, most of the cases showed neurological involvement (*N* = 21/21), including intellectual disability/developmental delay (*N* = 20/21), epilepsy (*N* = 21/21) and/or behavioral abnormalities (*N* = 8/21); meanwhile, only one case (familial, ET173; having a PV in *TSC1*) presented epilepsy without intellectual disability (Supplementary Table S1).

The presence of cardiac rhabdomyoma was observed in eight of the 22 above-described patients (36%), one with a PV in *TSC1* and the remaining seven with alterations in *TSC2*. In four of those cases, the rhabdomyoma presented complete regression (ET107, ET238, ET159, ET87), while the remaining four cases did not require medical or surgical management. In a single case (ET200), the rhabdomyoma was detected prenatally. Renal angiomyolipomas were identified by ultrasound in five cases (5/22; 23%), only one of which harbored a PV in *TSC1*. Case ET171 (harboring an LPV) was the only patient in our series that died during the study period; this occurred due to bronchopneumonia at 1 year 9 months of age. Variable expressivity could be corroborated in five out of six familial cases that had detailed TSC clinical information available and harbored a previously unreported PV (Supplementary Table S1). In the putative gonadal mosaicism case (ET28), the index case displayed a mild intellectual disability and epilepsy, while the brother reportedly exhibited psychotic episodes with moderate intellectual disability. In three cases with a novel PV, the parents showed multiple dental pits (mothers of ET117 and ET122, and father of ET243) or hypopigmented macules and learning disability (mother of ET201, who had an LPV). However, SS analysis led us to exclude minimal expression of the TSC phenotype.

We were unable to identify a PV, LPV or VUS in patients ET61, ET44 and ET223; interestingly, all three of them had epilepsy and cardiac rhabdomyomas that persisted at 17, 6 and 10 years of age, respectively and only one of them showed severe intellectual disability (ET223).

Overall, of the 66 cases studied herein, six presented with SEGA (ET22, ET41, ET56, ET104, ET277, ET278), three were *TSC2-PKD1* CGS cases (ET1, ET178, ET183) and one case showed an atypical large and bilateral fibrous cephalic plaque (ET159).

## Discussion

The clinical characterization of early-stage TSC has proven challenging due to the variable expressivity of the disease and the absence of any clear genotype-phenotype correlation. Most of the cases examined herein were diagnosed before 10 years of age (*N* = 51/66; 77%); this was similar to a previous study with a larger sample size (*N* = 197/243; 81%)^23^ performed at two different Hospitals in Boston, and there was no statistically significant difference in the age of diagnosis between the two studies (*P* = 0.48, Fisher’s exact test, 2-tailed). However, as only four of our TSC cases were diagnosed in the first 6 months of life, it could be useful for clinicians in Mexico to monitor specific clinical signs that have recently been reported to be useful for an earlier TSC diagnosis (before 6 months)^1,24^.

The emergence and routine implementation of new molecular techniques, such as MLPA and NGS, have revolutionized TSC diagnosis and increased the mutation detection rate to ~80–96%^12,14,15,25–29^. In this study, a PV or LPV was identified in 62 (94%) of the 66 included cases that fulfilled definitive TSC diagnosis criteria. Most of the 62 PV/LPV were present in *TSC2* (77% compared to 23% in *TSC1*) and there was a greater proportion of SV (90%) compared to CNV (10%). These data agree with the findings of multiple previous studies in other populations, which showed that the causative mutation rate was 77–85% in *TSC2* vs. 15–23% in *TSC1*, and that the mutations were 87–94% SV compared to only 6–13% large deletions^12,14,25,26^.

Regarding the mutational spectrum, frameshift and nonsense mutations were the most common variants in both genes (see Figure 2), whereas there were few intronic variants in *TSC1* or *TSC2* (7% vs. 10%, respectively). For CNV, large deletions were more common in *TSC2* than in *TSC1* (8%, N = 5/62 vs. 1.6%, N = 1/62), and showed proportions similar to those in other reports (5.6–7% vs. 0.5%)^5,30^. We did not find any other significant difference in the mutational spectrum between *TSC1* and *TSC2* (*P* > 0.5, Fisher’s exact test, 2-tailed Fig. 2d).

In terms of the genetic distributions of SV and CNV found in this study, four of the 13 SV found in *TSC1* (*N* = 4/13; 31%) were located in exon 15, which agrees with that reported in LOVD and various other publications (9.5–34%)^5,8,10,14,25^. This apparent accumulation of variants could be because exon 15 is the largest coding exon (559 bp) in *TSC1*. Four other SV were identified in exons 17 and 18, which form part of the coiled-coiled domain; together, these three exons (15, 17 and 18) presented the highest mutation frequency in *TSC1* (62%). Similarly, Hung *et al*.^31^ found that up to 89% of the identified PV localized to this region in Taiwanese TSC families. In the tuberin-binding domain (exons 10–13), in contrast, no PV was identified in our patients. There is debate as to whether this domain is a mutation region: some studies showed it to be a low-frequency mutation site^25,31–35^, while others found the opposite^5,14,15,36^. In *TSC2*, the GTPase-activating protein (GAP) binding domain (exons 35–39) contained 10 out of the 43 total SV (23%) identified herein, and the remaining were distributed throughout the gene.

In this study, recurrent PV were observed in both *TSC1* and *TSC2*. In *TSC1*, the frameshift p.(Lys630Glnfs*22) PV, which was previously reported as one of the most common mutations in that gene^10^, was seen in two of the 13 SV (15%) identified at this locus. In *TSC2*, the missense variants, p.(Ala614Asp) and p.(Pro1675Leu), were identified in two cases each (*N* = 2/43; 4.7%), while an in-frame microdeletion p.(His1746_Arg1751del) was seen in three cases (*N* = 3/43; 7%). The latter is the most frequently reported *TSC2* variant in the literature (*N* = 5/182, 2.8%; *N* = 4/98, 4.1%; *N* = 9/158, 5.7%)^25,36,37^ and could therefore be considered a potential hotspot. In this context, it is notable that we observed a novel microduplication affecting the same nucleotides and amino acids p.(His1746_Arg1751dup) in patient ET171. Our detailed analysis revealed that the microdeletion involved the CCG motif located three nucleotides upstream of the 5’ breakpoint and the microduplication involved the GTA motif located four nucleotides downstream of the 3’ breakpoint. These motifs are thought to favor replication slippage and are overrepresented in the close vicinity of microdeletions and/or microduplications^38^. Also, the ACTTAC motif located downstream of the 3’ breakpoint near the donor splice site, may promote secondary structure formation at the DNA level, increasing the potential for microdeletions and microinsertions^38^. Therefore, this region is prone to microdeletions (113 reported patients in LOVD: TSC2_00149) and microduplication (one case reported herein) due to its particular DNA architecture and could be considered a *TSC2* hotspot.

Regarding TSC inheritance, it is more often found sporadic cases (~85%) than familial ones^25^. We describe a similar proportion herein: 54 cases (82%) lacked any family history of TSC and 12 cases were familial (18%). When we examine only the *de novo* cases, which were defined as patients for whom the molecular study discarded the presence of a PV in either parent (*N* = 34), there were approximately four times more PV in *TSC2* than in *TSC1* (28 vs. 6 cases, respectively). In contrast, the familial cases showed similar proportions of PV in *TSC1* versus *TSC2* (5 vs. 7 cases; no significant difference, *P* = 0.1235 by Fisher’s exact test). These findings are comparable to previous reports that 67–85% of TSC cases were found to be caused by *de novo* germline mutations, mostly located in *TSC2*^14,15,25,39,40^ (two to ten times more often than in *TSC1*^14^). The familial cases showed no difference in the mutation frequency between *TSC1* and *TSC2*^14,25,36^, but Dabora *et al*.^25^ pointed out that the reported frequencies of *TSC1* and *TSC2* mutations in familial cases could be biased by the small number of families studied. Germline mosaicism was suggested in one of the familial cases (ET28) and even though germline mosaicism is rarely seen in TSC (6%) and we found a somewhat lower rate (1/66 cases; 1.5%), a conservative 2–3% recurrence risk should be advised for apparently sporadic TSC families^41^

Our search of the literature and public databases for previous reports of the 62 mutations found in *TSC1*/*TSC2* allowed us to determine that 25 (40%) of the PV/LPV found in the present work were novel, which was a higher proportion than those found in previous studies (38%, 29%, 22%) using Greek and Malaysian populations^15,26,32^. This is expected since this disease presents high allelic and locus heterogeneity, and emphasizes the importance of implementing multiple and diverse molecular techniques to evaluate coding and non-coding regions in both genes, and to discriminate SV from CNV. Our results are similar to those of Yu *et al*.^42^, who found a high percentage (54%) of new TSC variants but included a very limited number of cases (*N* = 11).

The molecular algorithms for detecting mutations in *TSC1* and *TSC2* by combining direct SS, NGS and MLPA techniques have been shown to achieve a very high mutation detection power^15,26,32^. Here, although we used a combined molecular methodology, there were three cases (ET44, ET61 and ET223) that fulfilled the criteria for a definitive TSC diagnosis but in whom no mutation was identified (NMI; 4.5%). Our NMI cases could have mutations in regions not covered by the SS and NGS techniques (promoters, regulatory regions and deep intronic mutations affecting splicing and branch point sites), mosaicism at a very low allelic frequency that could not be detected by the implemented bioinformatic algorithm and/or epigenetic modifications leading to transcriptional silencing^11^.

The protein modeling of the two missense LPV (cases ET201 and ET171) showed that these changes could induce potential structural alterations in important functional regions of the hamartin and tuberin proteins. In hamartin, the insertion of Lys112Glu113Val114 occurred at a potentially hydrophobic region. The residues were predicted to be buried in relatively rare hydrophobic cavities and would not be compatible with the hydrophobic interior of proteins^43,44^, and consequently would be likely to alter the structure and function of the protein. In tuberin, the introduction of HisIleLysArgLeuArg at C-terminal positions 1752–1757 appears very likely to alter the GAP domain. This region is important, since it regulates the GTP-binding domain and hydrolyzes Ras superfamily proteins that contribute to regulating cell growth regulation, proliferation and differentiation^5^. Moreover, it has been demonstrated that the C-terminal region of tuberin contains various important zones, including amino acids that are relevant for calmodulin binding (amino acids 1740–1757), a region that overlaps with estrogen receptor-α (amino acids 734–1807) and a nuclear localization signal (amino acids 1743–1755). All these regions are close to the amino acids that are inserted in our case, and their functions could potentially be affected.

Here, the intronic c.3815–21G>A variant was classified as a VUS. It was previously reported in human subject databases [e.g., dbSNP (rs778201014) and ExAC] at very low allelic frequencies (total AF = 0.0001279, Latino AF = 0.0009600) and with no homozygotes. At present, the actual effect of this variant is unknown. Caminsky *et al*.^45^ pointed out that the acceptor site (3′) of human consensus splice site sequences extends 26 nucleotides upstream from the exon boundary. The VUS identified herein is at the −21 position, prompting us to hypothesize that this genetic variant could have a deleterious impact on spliceosome recognition. Further functional studies are needed to corroborate the role of this VUS and the two missense LPV described above.

To date, it has proven difficult to establish any genotype-phenotype correlation in TSC syndrome. Some authors have proposed that *TSC2* mutations are associated with a more severe phenotype (early age of seizure onset, lower cognition index and the presence of subependymal nodules, SEGA, cardiac rhabdomyomas and/or renal angiomyolipomas)^14–16^. However, in other studies, the occurrence of tubers, seizures (*P* = 0.595) and (sub)cortical tubers (*P* = 0.299) did not differ between cases with a *TSC1* or *TSC2* mutation^14,16^. We were unable to determine a genotype-phenotype correlation from our cases that harbored novel PV, as all these genetic variants occurred only in one family. Most of these patients showed seizures and intellectual disability (*N* = 20/21; 95%) regardless of whether they harbored a PV in *TSC1* or *TSC2*; however, this feature could be biased because the study population was drawn from a tertiary referral hospital, where most of the cases show a severe condition. We found that cardiac rhabdomyomas and renal angiomyolipomas were more common in patients with a PV in *TSC2* than in *TSC1* (7:1 and 4:1, respectively); in this, our results are similar to those of other published studies^26,34,42^. Even though cardiac rhabdomyomas are the most common prenatal cardiac tumor related to TSC (50–86% of cases), the absence of other manifestations at this age makes it difficult to establish a definitive diagnosis^46^. Of the cases studied herein, only one case [ET200, with a novel PV in *TSC2* (c.1258–2A>G)] had prenatal detection of rhabdomyoma; however, the presence of hypomelanotic macules at neonatal age allowed for a definitive diagnosis of TSC. None of our patients presented any cardiac complication, which is consistent with the report that most of the rhabdomyomas in TSC ( > 60%) are asymptomatic^46^.

The NMI cases generally showed milder phenotypes (low severity and prevalence of seizures, less serious brain findings on imaging studies and better intellectual capacity) compared to those cases with a PV in *TSC2*^47^. In our NMI cases ET61 and ET223, epilepsy was reported at 2 and 9 years of age, respectively, but absent at 17 and 10 years of age, respectively. Two of the three NMI cases (ET61 and ET44) did not exhibited intellectual disability, whereas the third (ET223) had a clinically severe cognitive affliction. Finally, the three NMI cases had cardiac rhabdomyomas at 17, 6 and 10 years of age, respectively. This is relevant given that the majority of TSC patient were found to have partial (50%) or complete (18%) rhabdomyoma involution upon follow-up echocardiography^46^.

## Conclusion

Our combined molecular screening using SSCP/SS/MLPA/NGS reached a mutation detection rate of 94% and revealed a clear predominance of *TSC2* mutations and a majority of sporadic cases. Due to the great allelic and locus heterogeneity that exists in TSC and the large number of novel variants, it remains difficult to identify any genotype-phenotype correlation. This genetic study, however, enabled us to provide accurate genetic counseling, such as discarding minimal expression in first-degree relatives and defining familial versus sporadic cases. Our 3-D protein modeling results showed that the two missense LPV could alter the protein structure and function, but *in vitro* assays are needed to determine the real effects of these variants on the activities of hamartin and tuberin. Regarding the three cases with NMI, additional analyses are needed to rule out the presence of mosaicism or epigenetic *TSC1/TSC2* modifications. The fact that 40% were novel variants supports the importance of studying the genetics of different TSC populations in order to expand our knowledge of the genetic spectrum of this disease, both worldwide and in countries such as Mexico, where molecular studies are limited and little work has been done on this disease. Therefore, this work represents the first TSC molecular screening performed in our country.

## Methods

### Genomic DNA extraction and PCR

All patients have a statement attesting to the informed consent of a parent and/or legal guardian for participation in the study and their parents signed their written informed consent. The study was conducted in accordance with the Declaration of Helsinki and Institutional Review Board (Comité de Ética en Investigación, Instituto Nacional de Pediatría, México) approval was obtained (protocol reference number 060/2014). Total peripheral blood leukocytes or buccal swab cells were obtained from the 66 cases, their available parents and first-degree affected family members. Genomic DNA was obtained with a commercially available kit using a silica-based approach (QIAamp; Qiagen, Victoria, Australia) according to the manufacturer’s protocol. Specific primers were designed to enable PCR amplification of coding regions and intron-exon boundaries (±20 base pairs) of the *TSC1* (NG_012386.1, NM_000368.4) and *TSC2* (NG_005895.1, NM_000548.3) genes. Primer sequences and amplification conditions are available upon request.

### Single-strand conformation polymorphism

All *TSC1* and *TSC2* PCR fragments were subjected to SSCP analysis. Briefly, 9 µL of denaturing solution (0.05% w/v bromophenol blue, 0.25% xylene-cyanol, 1.17 M sucrose and 5 M urea) was mixed with 5 µL of PCR product, heated for 10 min at 94 °C and chilled on ice. Samples (2.5–25 ng) were loaded on a 1X polyacrylamide gel prepared according to the manufacturer’s protocol (MDE, Lonza, Rockland, USA). Electrophoresis was performed at 25 W for 5 h; the temperature was kept constant (4^o^C) through cold-water circulation. The gel was stained with silver nitrate solution according to the manufacturer’s protocol (Silver Stain Plus kit; Bio-Rad).

### Sanger sequencing

Samples displaying an abnormal SSCP migration pattern (two bands with different electrophoretic mobilities) were subsequently sequenced using an automated bidirectional Sanger method applied by Macrogen, USA (Rockville, Maryland, USA). For the five cases in which SS was the initial molecular study, PCR amplification products for all coding exons and intron-exon boundaries (±20 base pairs) of *TSC1* and *TSC2* were subjected to automated bidirectional Sanger sequencing (performed by Macrogen, USA). The obtained electropherograms were aligned to reference *TSC1* and *TSC2* gene sequences (NG_012386.1 and NG_005895.1, respectively) and posteriorly analyzed with the Codoncode Aligner software (CodonCode Corporation, Dedham, MA, USA) to detect small variants (point mutations and small insertions, deletions or duplications). In addition, the clinically relevant variants identify by NGS of coding and exon-intron boundaries (±50 base pairs) sequences were confirmed by SS for index cases and first-degree relatives.

The Mutalyzer nomenclature module tool (http://www.mutalyzer.nl) was used to validate the sequence variant nomenclature of all the *TSC1* and *TSC2* variants reported herein according to the guidelines of the Human Genome Variation Society. The novel variants have been submitted to LOVD v.3.0. (for accession numbers, see Table 1).

### In-silico evaluation tools

The three likely pathogenic variants [LPV; p.(Leu112_Leu113delinsLysGluVal), p.(His1746_Arg1751dup), c.737+3A>G] and the variant of uncertain significance (VUS; c.3815–21G>A) were subject to *in silico* evaluation using different bioinformatics tools under default parameters. The following tools were used: PROVEAN Score (http://provean.jcvi.org/seq_submit.php) and Mutation Taster (http://www.mutationtaster.org/) for the missense LPV; and Splice Site Finder (http://www.umd.be/HSF/), MaxEntScan (http://hollywood.mit.edu/burgelab/maxent/Xmaxentscan_scoreseq.html), NNSPLICE (https://www.fruitfly.org/seq_tools/splice.html) and GeneSplicer (https://ccb.jhu.edu/software/genesplicer/) for intronic variants.

### Multiplex ligation-dependent probe amplification (MLPA)

Copy number variants (CNV) in *TSC1* and *TSC2* were assessed with the MLPA technique using SALSA MLPA P124-C1 probemix for *TSC1* and P337-A2 for *TSC2* (MRC-Holland Amsterdam, The Netherlands). Amplified products were posterior analyzed by electrophoresis on an Applied Biosystems 3500 Genetic Analyzer (Thermo Fisher Scientific, USA). Comparative data analysis was performed with the Coffalyser.Net (v.140701.0000) software (MRC-Holland Amsterdam, The Netherlands).

### Next-generation sequencing and data analysis

DNA libraries were prepared with KAPA Hyper Prep (Kapa Biosystems, Inc. Wilmington, MA, USA), following the manufacturer’s protocol. *TSC1* and *TSC2* exons and intron boundaries (±150 bp) were captured by hybridization with 125-mer probes for 30 nucleotides with 50x tiling (designed by Twist Bioscience, San Francisco, CA, USA) for the hg38 reference genome. Captured DNA was sequenced on the Illumina HiSeq. 2 × 150 Platform for an 800x mean coverage, as performed by Admera Health Company (South Plainfield, NJ, USA). The raw sequencing data were evaluated for quality with the FastQC program (Version 0.11.8)^48^. Adapters and low-quality reads (Phred value <20) were excluded with the Trimmomatic v 0.35 software^49^. Filtered reads were aligned with Bowtie2^50^ against human genome version GRCh38, and optical and PCR duplicates were removed by SAMtools^51^. Single nucleotide variants were detected with the GATK^52^ and FreeBayes^53^ programs and posteriorly annotated with GATK^52^.

Once the responsible TSC genotypes were determined as described above, the Fisher’s exact test was used to compare the proportions of different *TSC1* and *TSC2* gene variants (Fig. 2e).

### Protein modeling of N-term TSC1 and C-term TSC2 proteins

The amino acid sequences of hamartin N-term (amino acids 1–200; hamartin isoform X1 [Homo sapiens] NCBI: XP_011517281.1) and tuberin C-term (amino acids 1021–1807; tuberin isoform 1 [Homo sapiens] NCBI: NP_000539.2) were obtained from the NCBI databases, and the ITASSER (https://zhanglab.ccmb.med.umich.edu/I-TASSER/)^54–56^ server was used to perform homology modeling. The mutants, hamartin 111–112LL/KEV (insertion/deletion) and tuberin 1747–1752-IKRLRH (insertion), were also modeled. The obtained models were subjected to energy minimization with the YASARA software^57^ and quality validation with MolProbity^58^. Finally, the predicted 3D structures were modeled with PyMOL^59^ (http://www.pymol.org).

## Supplementary information

Supplementary information.

## Data Availability

Twenty-five pathogenic or likely pathogenic variants, not previously reported, were submitted and are available at the Leiden Open Variation Database; LOVD (www.lovd.nl/TSC1 and www.lovd.nl/TSC2).
