# Supplementary material for: First comprehensive TSC1/TSC2 mutational analysis in Mexican patients with Tuberous Sclerosis Complex reveals numerous novel pathogenic variants
Source: Sci Rep. 2020 Apr 20;10:6589. doi: 10.1038/s41598-020-62759-5 (PMC7170856; doi:10.1038/s41598-020-62759-5)
Supplement: Supplementary file 1 — Supplementary information. [file 41598_2020_62759_MOESM1_ESM.pdf]

First comprehensive *TSC1*/*TSC2* mutational analysis in Mexican patients with Tuberous Sclerosis Complex reveals numerous novel pathogenic variants

Miriam E. Reyna-Fabián[1], Nancy L. Hernández-Martínez[1], Miguel A. Alcántara-Ortigoza[1], Jorge T. Ayala-Sumuano[2], Sergio Enríquez-Flores[3], José A. Velázquez-Aragón[1], Alfredo Varela-Echavarría[4], Carlos G. Todd-Quiñones[5,6] & Ariadna González del-Angel[1]*.

[1]Laboratorio de Biología Molecular, Departamento de Genética Humana, Instituto Nacional de Pediatría, Secretaría de Salud, Ciudad de México, México.
[2] IDIX SA de CV. Querétaro, México.
[3]Grupo de Investigación en Biomoléculas y Salud Infantil, Laboratorio de Errores Innatos del Metabolismo y Tamiz, Instituto Nacional de Pediatría, Ciudad de México, México.
[4]Departamento de Neurobiología del Desarrollo y Neurofisiología, Instituto de Neurobiología, Universidad Nacional Autónoma de México, Querétaro, México.
[5]Posgrado en Biología Experimental, Universidad Autónoma Metropolitana-Iztapalapa, México D.F., México.
[6]Departamento de Genética Humana, Laboratorio de Biología Molecular, Hospital de Alta Especialidad de Veracruz, SESVER.

*Correspondence to ariadnagonzalezdelangel@gmail.com

Supplementary Information

Supplementary Table S1. Clinical characteristics of 21 cases with a novel pathogenic variant (PV), two cases with a likely pathogenic variant (LPV) and protein modeling analysis, one case with a variant of uncertain significance (VUS) and three patients with no mutation identified (NMI). **Abbreviations**. AF: angiofibromas (Ó3); AML: angiomyolipomas (Ó2); CD: cortical dysplasia; CR: cardiac rhabdomyoma; DEP: dental enamel pits (>3); dN: *de novo*; f: familial; F: feminine; FCP: fibrous cephalic plaque; HM: hypomelanotic macules (Ó3); IOF: intraoral fibromas (Ó2); LAM: lymphangioleiomyomatosis; m: months; M: masculine; MRH: multiple retinal hamartomas; RAP: retinal achromic patch; SEGA: subependymal giant cell astrocytoma; SEN: subependymal nodules; SN: suspected *de novo*; SP: shagreen patch; UF: ungual fibromas (Ó2); y: years; ¿? No parental DNA available for testing; +: features present at the time of diagnosis or during medical follow-up; ♣: extensive fibrous cephalic plaque; ♦: pneumocytic micronodular multifocal hyperplasia. ♠: died at 1 year 9 months attributed to bronchopneumonia. **Notes**. Confetti skin lesions, multiple renal cysts and non-renal hamartomas (minor features) are not shown because they were not presented by any of the investigated patients. Detailed clinical information was not available for cases ET146 and ET90.

									Major features											Minor features		
Gene	Case	Variant	Sex	Age at diagnosis	Inheritance	Seizures	Intellectual disability	Behavioral disorder	HM	AF/FCP	UF	SP	MRH	CD	SEN	SEGA	CR	LAM	AML	DEP	IOF	RAP
**PV identified in *TSC1* and *TSC2***																						
TSC1	ET173	c.89_102del14	M	12y	F	+			+	+		+			+					+		
	ET157	c.1458_1461del	M	6y	SN	+	+	+	+	+					+							
	ET107	c.2101C>T	M	2y	dN	+	+		+	+				+	+		+		+			
	ET117	c.2596_2600dup	M	9y	dN	+	+	+	+	+	+	+		+	+					+	+	
	ET254	Deletion Ex 15-23	F	7y	dN	+	+	+	+	+												
TSC2	ET166	c.668dup	M	6y	SN	+	+	+	+	+				+	+							
	ET41	c.1258-1G>C	M	10y	dN	+	+		+	+		+	+	+	+	+	+					
	ET200	c.1258-2A>G	M	1m	¿?				+								+					
	ET32	c.1881_1882dupCC	F	1y	dN	+	+		+	+				+	+				+			
	ET238	c.2172dup	M	3y	dN	+	+	+	+	+				+	+		+					
	ET53	c.2309_2315del	F	8m	dN	+	+		+	+		+			+							
	ET122	c.2448dup	F	3y	SN	+	+		+	+			+	+						+	+	
	ET159	c.2640-1G>T	F	2y	SN	+	+			♣			+	+	+		+	♦	+			+
	ET278	c.3134_3136delinsTTTT	F	7m	SN	+	+		+					+	+	+	+					
	ET243	c.3179G>C	M	5y	dN	+	+		+						+							
	ET87	c.3277G>T	M	4y	dN	+	+	+	+	+				+	+		+		+			
	ET175	c.3371_3381del	M	11y	F	+	+			+							+					
	ET145	c.3538A>T	F	2m	F	+	+	+		+			+	+	+							
	ET28	c.3624G>A	F	14y	F	+	+		+					+	+							
	ET168	c.4367_4385del	M	6y	F	+	+		+					+	+							
	ET16	c.4560delG	F	9y	dN	+	+		+	+		+		+			+					
	ET114	c.4849+2_4849+11del	M	3y	SN	+	+	+	+	+		+			+		+			+		
**LPV and VUS identified in *TSC1* and *TSC2***																						
TSC1	ET201	c.333_337delinsAAAAGAGG (LPV)	M	7y	SN	+			+						+							
	ET171♠	c.5238_5255dup (LPV)	M	7m	SN	+	+							+	+		+					
TSC2	ET81	*TSC2* c.3815-21G>A (VUS)	M	8y	SN	+	+		+	+		+		+						+	+	
	ET61	(NMI)	M	7y		+			+	+		+	+		+		+		+			
	ET44	(NMI)	F	2y		+		+	+	+					+		+					
	ET223	(NMI)	F	11y		+	+			+	+	+		+	+		+			+	+	
